# Long-term efficiency of cataract surgery with hydrophilic acrylic Ioflex intraocular lens

**DOI:** 10.6061/clinics/2017(09)04

**Published:** 2017-09

**Authors:** Priscilla A. Jorge, Camila Ribeiro Koch, Delano Jorge, Newton Kara-Junior

**Affiliations:** I Departamento de Oftalmologia, Faculdade de Medicina FMUSP, Universidade de Sao Paulo, Sao Paulo, SP, BR; II Departamento de Oftalmologia, Universidade Federal de Sao Paulo, Sao Paulo, SP, BR

**Keywords:** Cataract, Lenses, Intraocular, Economics, Postoperative Complications, Cost and Cost Analysis

## Abstract

**OBJECTIVE:**

To evaluate the efficiency of long-term cataract surgery using low-cost intraocular lens implantation in community campaigns.

**METHODS:**

Fifty-eight randomly selected patients were evaluated four years after phacoemulsification and Ioflex intraocular lens implantation. Causes of low visual acuity related to the intraocular lens were evaluated, and treatment costs were calculated.

**RESULTS:**

The mean age of patients was 72±10.2 years. Four years after surgery, 25 eyes (43.0%) had decreased visual acuity related to the intraocular lens: posterior capsule opacification was noted in 24 eyes (41.3%), and intraocular lens opacification was noted in one eye (1.7%). The total cost of the post-surgical complication treatments represented 6.3% of the initial budget of the entire surgical patient group.

**CONCLUSIONS:**

The efficiency of cataract surgery with low-cost Ioflex intraocular lens implantation was significantly reduced in a long-term follow-up study because postoperative complications related to intraocular lenses emerged at higher rates than when the gold-standard treatment was used.

## INTRODUCTION

Phacoemulsification surgery is the most widely used technique for cataract surgery due to its good visual outcomes 1. However, long-term postoperative complications due to different intraocular lens (IOL) materials and designs can affect budgets and impair the efficiency of implementing this procedure in public healthcare systems 2,3. Given the large number of cataract surgeries being conducted, even small differences in costs per surgery add up to larger amounts in total cost to the healthcare system 4.

Ioflex (Mediphacos, Belo Horizonte, MG, Brazil) is a low-cost hydrophilic acrylic IOL commonly used in community programs for cataract surgery for underprivileged people 2. This IOL has been marketed for more than 15 years and has been approved by the national health agencies in several countries in Europe, Asia, and Latin America. An efficient IOL should produce good results with low cost. Reports indicate post-surgical complications related to the Ioflex IOL 2,5 and suggest that health managers must base their actions on clinical evidence of its efficiency 6. Thus, for the first time, this study evaluates the long-term efficiency of cataract surgery using the Ioflex IOL.

## MATERIALS AND METHODS

A sample of 102 subjects was randomly selected from a population of 192 patients who underwent cataract surgery and Ioflex IOL implantation within the past four years without intraoperative complications as part of a community campaign for underprivileged people in Pernambuco, Brazil. The follow-up time ranged from 42 to 48 months postoperatively. The mean follow-up time was 45.6 months. A total of 31 patients could not be located due to either communication difficulties related to geographical distance or patient death. Of the 71 patients located, 13 (18.3%) were not present for examination. Thus, 58 patients were available for examination. To avoid bias, only the right eye was included in the study for patients who underwent cataract surgery in both eyes.

The same surgeon performed all surgical procedures. In each case, a 2.8-mm incision was made under peribulbar anesthesia using a balanced salt solution (BSS^®^, Alcon Labs, Fort Worth, TX, USA) and 2% methylcellulose viscoelastic (Ophthalmus, São Paulo, Brazil). Continuous curvilinear capsulorhexis approximately 1 mm smaller than the optical part of the IOL and cortical clean-up procedures were used in all cases. Data were collected from the medical records and included information about the patient’s ocular health, pre-surgical corrected visual acuity (VA), and postoperative corrected VA after one month. Examination of the corrected VA, a biomicroscopy slit lamp examination, and fundoscopy were performed at the evaluation four years after the surgery. Posterior capsule opacification (PCO) was diagnosed during the slit lamp examination, and the decision regarding laser capsulotomy was based on VA deterioration. Patients with worsened VA (one line or more on Snellen card notation) compared to that measured one month after surgery, without improvement with glasses, that was related to PCO were referred for neodymium:yttrium-aluminum-garnet (Nd:YAG) laser treatment.

Surgical and post-surgical procedure costs were based on Brazilian Health System guidelines. All values were converted to US dollars (USD) at a rate of one dollar to R$ 3.1 (Brazilian currency).

The postoperative complications of the IOL were compared with those of a gold-standard hydrophobic IOL (AcrySof SN60 one-piece, Alcon, Fort Worth, TX, USA) based on a study by Cullin et al. 7, who evaluated PCO rates and related costs for three different IOLs in a retrospective study of 1,527 patients with a mean follow-up time of 41.5 months, ranging from 19 to 76 months. The mean follow-up time of patients with the hydrophobic AcrySof IOL was 41.4 months. The study included Nd:YAG capsulotomy patients with development of postoperative PCO and clinical signs of secondary visual impairment, such as reduced VA or glare.

Both our study and that by Cullin et al. 7 detected PCO at an outpatient visit. The criterion that we used for PCO was visual loss after primary cataract surgery as subjective data to suggest the necessity of Nd:YAG laser capsulotomy. In addition, none of the studies described the use of Trypan blue during the surgeries.

### Ethics

This study was approved by the Ethics Committee of the Altino Ventura Foundation (054/2011) in accordance with the Helsinki Declaration.

## RESULTS

The mean age of the patients who received an Ioflex IOL was 72.0±10.2 years. The mean preoperative corrected VA was 0.79±0.41 logMAR (20/125 in Snellen card notation). One month after surgery, the mean corrected VA improved to 0.25±0.20 logMAR (20/35 in Snellen card notation), except for one patient who experienced a posterior segment problem. Four years after surgery, 39 (67.0%) of the 58 evaluated eyes had detectable PCO upon biomicroscopy evaluation, and 24 eyes (41.3%) had decreased VA due to PCO. Patients with slight PCO underwent a new refractive examination for updated glasses and exhibited a mean corrected VA of 0.13±0.03 logMAR (20/26 in Snellen card notation), whereas the mean corrected VA of the 24 patients with decreased VA due to PCO was 0.53±0.21 logMAR (20/67 in Snellen card notation). These patients were referred for Nd:YAG laser capsulotomy and consequently demonstrated a corrected VA that had recovered to 0.05±0.06 logMAR (20/22 in Snellen card notation). One patient (1.7%) exhibited decreased VA from 0.09 logMAR at one month after surgery to 0.6 logMAR 4 years after surgery (20/25 to 20/80 in Snellen card notation). This result was due to IOL opacification, and the patient was referred for IOL exchange, resulting in a final corrected VA recovery to 0 logMAR (20/20 in Snellen card notation). Two additional patients with PCO were diagnosed with glaucoma and posterior segment disease.

The cost of each Ioflex IOL was USD $50.00, and the cost of each gold-standard hydrophobic IOL was USD $99.59. The costs of the cataract surgery, Nd:YAG laser capsulotomy procedures, and IOL exchange are listed in Table 1. The additional cost to maintain a good visual outcome 4 years after surgery was USD $9.03 per patient with Ioflex IOL implantation, whereas the estimated cost of a gold-standard IOL implant would have been USD $1.07 per patient. Thus, the low-cost IOL is USD $7.96 greater than the gold standard IOL in the initial surgery budget (5.5%). The cost of the post-surgical procedures using the Ioflex IOL represents 6.3% (USD $524.00) of the initial budget of the entire surgical patient group.

## DISCUSSION

The results revealed high rates of late postoperative complications related to the IOL, with significant PCO in 41.3% of patients and IOL opacification in 1.7% of patients who underwent Ioflex hydrophilic acrylic IOL implantation. For the gold-standard hydrophobic acrylic IOL, 7.4% of patients had PCO, and no IOL opacification was reported 7. Hydrophilic acrylic lenses are associated with higher rates of PCO than hydrophobic IOLs.

Few papers report PCO evaluation more than four years after surgery. Ronbeck et al. 8 reported that hydrophobic acrylic IOLs delayed PCO development, with a median time of survival without Nd:YAG capsulotomy of 108 months. PCO rates were reported for approximately 50% of the patients approximately 10 years after surgery. We found similar rates approximately 4 years after surgery. Chang et al. 9 reported a rate of 10% for Nd:YAG laser capsulotomy for a one-piece hydrophobic IOL and 22% for a 3-piece hydrophobic IOL 5 to 7 years after surgery. These results demonstrate that hydrophobic acrylic IOLs may delay the need for laser capsulotomy for several years in eyes submitted to cataract surgery and exhibit lower occurrence rates than hydrophilic acrylic PCO rates. Given that the life expectancy of people worldwide is increasing, it is important to delay the occurrence of postoperative complications as much as possible.

Vasavada et al. 10 reported Nd:YAG laser capsulotomy cases in 12.9% and 16% of the hydrophilic IOLs evaluated and none in the hydrophobic group 3 years after surgery. Kugelberg et al. 11 reported capsulotomy in 42% of a hydrophilic IOL group compared to 10% in a hydrophobic group at 2 years after surgery. Gauthier et al. 12 reported an increasing number of Nd:YAG laser treatments over time, with 8.8% in the hydrophobic IOL group and 37.2% in the hydrophilic lens group with a follow-up of 24 months. The results from these studies are consistent with our results. This information could provide a basis for discussions about the long-term economic consequences of choosing an IOL with a design that affects the risk of PCO development.

It is not yet known whether the occurrence of PCO in hydrophobic IOLs is only 50% or continues to increase after 12 years post-surgery 8. Similarly, it is not known whether hydrophilic IOLs will continue to exhibit greater PCO rates than hydrophobic IOLs. More studies are needed to better explain PCO evolution. The results of our study allows us to estimate the future trends to at least better prepare managers and public health programs.

We have previously reported the occurrence of Ioflex IOL opacification, which exhibited and incidence rate of 7% 5. The current finding of a 1.7% IOL opacification rate was due to the selection criterion, which included only one eye of each patient. This criterion was not used in previous research. Although opacification is not a common complication, it is the main cause of hydrophilic acrylic IOL extraction. The causes of this complication are not completely understood, but the literature indicates that it is caused by calcification due to calcium and/or phosphate in the IOL 13.

Due to technological advances, the IOL design has been modified from a round edge to include a square edge, which has resulted in a reduction in the incidence of PCO 12. However, studies suggest that square-edged IOLs should completely encompass the 360 degrees around the IOL optic to provide an effective barrier 14. Furthermore, another study demonstrated differences between brands of square-edged lenses, suggesting that variations in their edge profiles may account for clinical differences in postoperative PCO rates 15. Acrylic IOLs also appear to lose their PCO preventive effect despite their sharp optic edges 8,16.

Our study suggests that the initial savings of USD $49.59 per patient with the use of the hydrophilic IOL compared to the hydrophobic IOL is reduced when considering the additional cost of treatments for avoidable postoperative complications. We estimate that the Ioflex IOL loses its efficiency, as it costs USD $7.96 more than the gold standard IOL in the initial surgical budget (5.5%). However, even with the additional cost of postoperative treatments, the total cost was still lower when using the low-cost IOL.

Community cataract programs are performed worldwide in developing countries 2,17. Reviews of blindness in regions such as Latin America, Nigeria, Cameroon, and India reported a need to improve the implementation of social action programs to prevent blindness and improve cataract surgery outcomes 18-23. In a long-term postoperative follow-up study, De Senne et al. 24 demonstrated that 75.6% (Project A) and 64.7% (Project B) of patients experienced low VA due to postoperative complications in a community cataract program in Brazil (mainly PCO and refractive errors). This finding indicates that long-term post-surgical complications can be as harmful as failing to have the cataract extracted.

Data from this study highlight the need for community cataract programs to provide easy access to specialized services over a long follow-up period to ensure the initial efficiency of the cataract treatment. This notion is particularly important when hydrophilic acrylic IOLs are used, especially the Ioflex IOL, which is widely used in community campaigns in Brazil given its low cost. Long-term postoperative complications pose a risk for the efficiency of cataract treatment. This risk could be partially avoided by using better IOLs with proven clinical results given that the majority of post-surgical complications with poor visual outcomes are related to low-cost IOLs 2,25.

This study revealed that use of the Ioflex acrylic hydrophilic IOL may cause the public health system to spend almost 6.3% more than the initial budget on post-surgical treatments. Therefore, the results of this paper should make managers aware that they should schedule an outpatient visit at least 4 years after surgery if the low-cost hydrophilic IOL is used and reserve a budget for Nd:YAG laser capsulotomy. In contrast, for hydrophobic IOLs, the visit could be scheduled 10 years after surgery.

The current study had some important limitations. The comparison was performed with another study, specifically a retrospective study with a different population and different surgeons. The follow-up time of PCO evaluation was similar but was not identical. None of the studies determined a criterion for software for image analysis to evaluate the severity of PCO. However, we believe that despite this limitation, the results were interesting and worth reporting.

In conclusion, the material and design of the Ioflex IOL afford it short-term effectiveness, but reduced efficiency for long-term cataract surgery is noted upon follow-up. After surgery, an increased number of patients underwent laser capsulotomy in a shorter period of time than with the high-cost IOLs. We have highlighted the social impact of a cost-effective IOL, particularly for public healthcare policies in developing countries.

## AUTHOR CONTRIBUTIONS

Jorge PA provided substantial contributions to the conception and design of the study, acquisition, analysis and interpretation of data, drafting and critical revision of the manuscript for important intellectual content and approval of final version of the manuscript. Koch CR provided substantial contributions to the conception and design of the study, drafting of the manuscript and approval of the final version of the manuscript. Jorge D provided substantial contributions to the acquisition of data, critical revision of the manuscript for important intellectual content and approval of the final version of the manuscript. Kara-Junior N provided substantial contributions to the conception and design of the study, analysis and interpretation of data, drafting and critical revision of the manuscript for important intellectual content and approval of the final version of the manuscript.

## Figures and Tables

**Table 1:** Surgical and post-surgical procedures expenses refund based on Brazilian Health System guidelines on the 58 eyes evaluated.

	Value/unit USD $	*N* of Procedures	Total cost USD $
Cataract surgery	142.90	58	8,288.20
Nd:YAG laser	14.51	24	348.24
IOL exchange	175.76	1	175.76
